# Distinct expression of CDCA3, CDCA5, and CDCA8 leads to shorter relapse free survival in breast cancer patient

**DOI:** 10.18632/oncotarget.24059

**Published:** 2018-01-09

**Authors:** Nam Nhut Phan, Chih-Yang Wang, Kuan-Lun Li, Chien-Fu Chen, Chung-Chieh Chiao, Han-Gang Yu, Pung-Ling Huang, Yen-Chang Lin

**Affiliations:** ^1^ Graduate Institute of Biotechnology, Chinese Culture University, Taipei, Taiwan; ^2^ NTT Institute of Hi-Technology, Nguyen Tat Thanh University, Ho Chi Minh City, Vietnam; ^3^ Department of Biochemistry and Molecular Biology, Institute of Basic Medical Sciences, College of Medicine, National Cheng Kung University, Tainan, Taiwan; ^4^ School of Chinese Medicine for Post-Baccalaureate, I-Shou University, Kaohsiung, Taiwan; ^5^ Department of Physiology and Pharmacology, West Virginia University, Morgantown, WV, USA; ^6^ Department of Horticulture & Landscape Architecture, National Taiwan University, Taipei, Taiwan

**Keywords:** cell cycle division-associated (CDCA) protein, breast cancer, cell cycle, prognosis, bioinformatics

## Abstract

Breast cancer is a dangerous disease that results in high mortality rates for cancer patients. Many methods have been developed for the treatment and prevention of this disease. Determining the expression patterns of certain target genes in specific subtypes of breast cancer is important for developing new therapies for breast cancer. In the present study, we performed a holistic approach to screening the mRNA expression of six members of the cell division cycle-associated gene family (CDCA) with a focus on breast cancer using the Oncomine and The Cancer Cell Line Encyclopedia (CCLE) databases. Furthermore, Gene Expression-Based Outcome for Breast Cancer Online (GOBO) was also used to deeply mine the expression of each CDCA gene in clinical breast cancer tissue and breast cancer cell lines. Finally, the mRNA expression of the CDCA genes as related to breast cancer patient survival were analyzed using a Kaplan-Meier plot. CDCA3, CDCA5, and CDCA8 mRNA expression levels were significantly higher than the control sample in both clinical tumor sample and cancer cell lines. These highly expressed genes in the tumors of breast cancer patients dramatically reduced patient survival. The interaction network of CDCA3, CDCA5, and CDCA8 with their co-expressed genes also revealed that CDCA3 expression was highly correlated with cell cycle related genes such as CCNB2, CDC20, CDKN3, and CCNB1. CDCA5 expression was correlated with BUB1 and TRIP13, while CDCA8 expression was correlated with BUB1 and CCNB1. Altogether, these findings suggested CDCA3, CDCA5, and CDCA8 could have a high potency as targeted breast cancer therapies.

## INTRODUCTION

According to the WHO report, the top five cancers were lung (1.69 million), liver (788,000), colorectal (774,000), stomach (754,000), and breast (571,000) (WHO, Fact Sheet, Feb 2017). Although the underlying mechanism of cancer development was extensively studied, breast cancer patient, particularly women, are still coping with low survival rate. Therefore, novel and effective therapeutic treatments and drugs development are very crucial.

Cell division is the critical process of life. Many studies have proven that a malfunction in the cell division process results in cancer [1–5]. The cell cycle division-associated (CDCA) protein family is comprised of eight members (CDCA1-8). Cell division cycle-associated protein 1 (CDCA1) is critical for nuclear division and microtubule stabilization [6]. The function of CDCA2 is binding to the protein phosphatase 1 γ (PP1γ) and controlling the DNA damage response in the cell cycle [7, 8]. CDCA3 is known to regulate cell cycle progression, and its levels are controlled by transcription and protein degradation during the G1 checkpoint of the cell cycle [9]. CDCA4 is a cell-cycle regulator that is associated with the G1/S transition [10]. CDCA4 also modulates p53 expression levels [11]. CDCA5 is critical regulator of sister-chromatid cohesion and separation during cell division [12]. CDCA7 is activated in hematopoietic stem cell precursors in the mouse embryo and maintained thereafter in distinct undifferentiated hematopoietic populations. CDCA8 is an essential regulator of mitosis [13].

The purpose of this study was to systematically investigate the relationship between the mRNA expression of the CDCA family and the survival probability of breast cancer patients using the Oncomine database (www.oncomine.org), Gene expression-based Outcome for Breast cancer Online database (GOBO; http://co.bmc.lu.se/gobo/gsa.pl), the Cancer Cell Line Encyclopedia database (CCLE; www.portals.broadinstitute.org/ccle), and a Kaplan-Meier plot (www.kmplot.com).

## RESULTS

### Expression of CDCA gene family in breast cancer tissue

**Figure 1:**
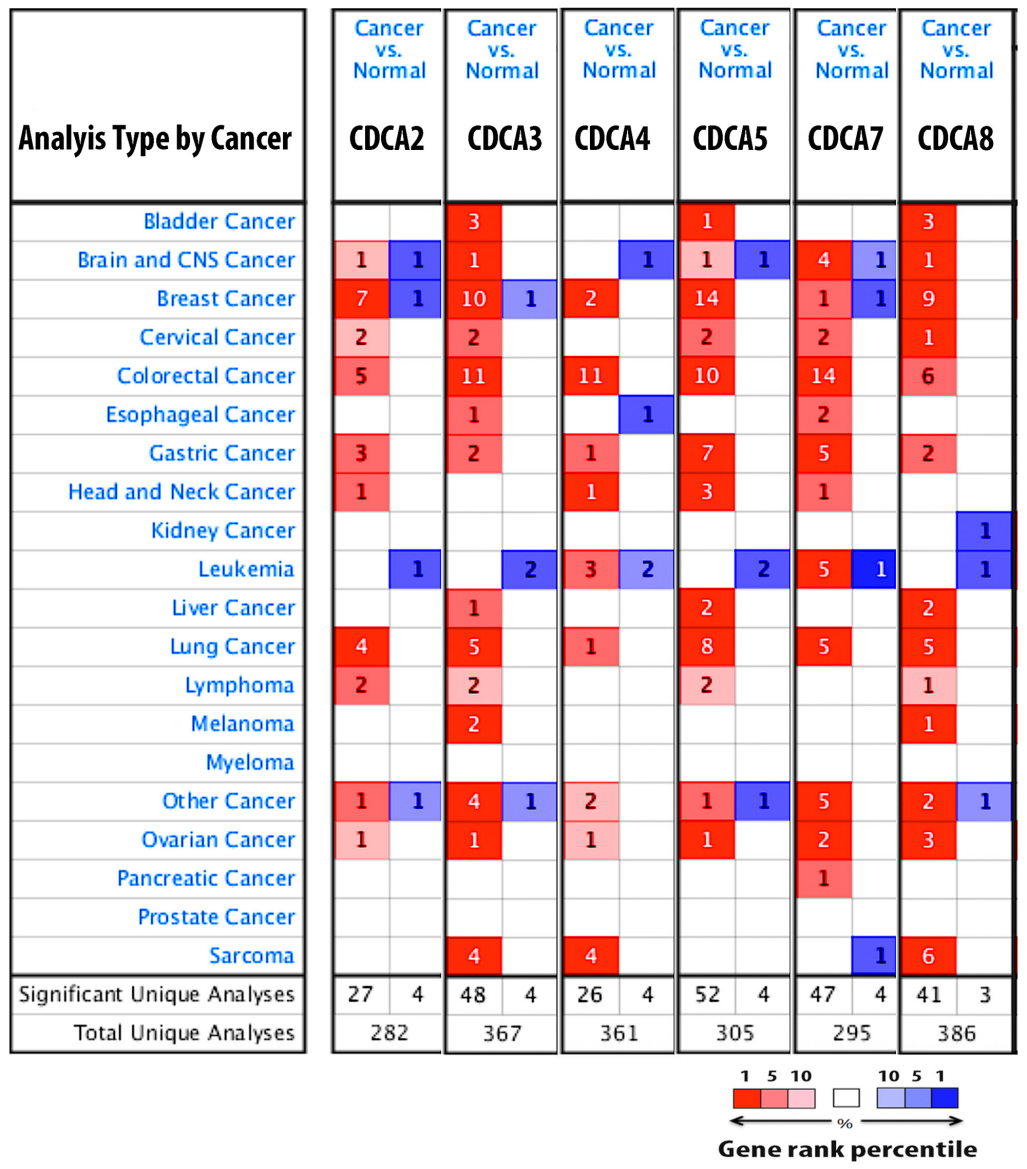
CDCA genes expression in 20 different types of cancer disease Data compared mRNA expression of gene in cancer tissue relative to normal matched type tissue. Over and under expression of CDCA genes were displayed with color based manner. Red color represents for over expression of gene while blue color is for under expression of gene. Color transparency slightly shifted top 1% to top 10% in bother over and under expression of gene. The number in each square denoted number of analyse(s) satisfy the threshold such as gene rank percentile (10%), p-value (10E-4), and fold change (1.5).

The expression of CDCA2, CDCA3, CDCA4, CDCA5, CDCA7, and CDCA8 in 20 types of cancer is depicted in Figure 1. CDCA genes were dramatically overexpressed in breast cancer tissue relative to normal type-matched tissue.

**Figure 2:**
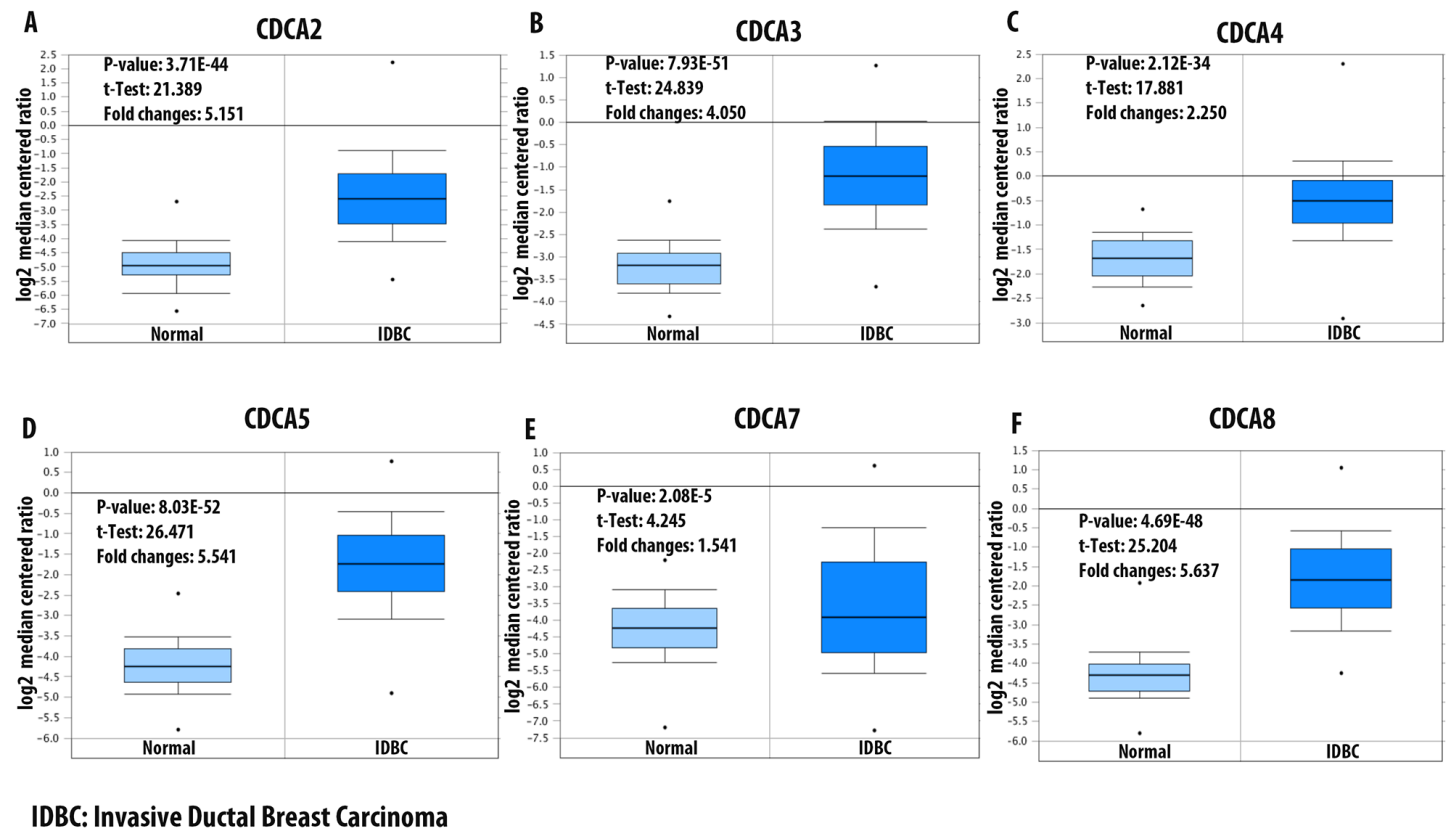
mRNA expression of CDCA family members in invasive ductal breast carcinoma (IDBC) Log2 median centered ratio was used to express the fold changes between CDCA genes in IDBC tissue relative to normal matched type tissue. P-value, *t*-test, and fold changes information were displayed. Panel **(A-F)** represent for CDCA2, CDCA3, CDCA4, CDCA5, CDCA7, and CDCA8 mRNA expression in IDBC relative to control samples.

Data mining from The Cancer Genome Atlas (TCGA) dataset for breast cancer showed significant overexpression of CDCA2 in some subtypes of breast cancer, which were Erb-B2 Receptor Tyrosine Kinase 2/Estrogen receptor/progesterone receptor (ERBB2/ER/PR) negative (triple-negative breast cancer [TNBC]), invasive ductal breast carcinoma, invasive breast carcinoma, and invasive lobular breast carcinoma. The invasive ductal breast carcinoma subtype had highest expression-level with an over five-fold higher expression in cancerous tissue compared to normal breast tissue (Figure 2A). Analysis of the TCGA breast dataset showed a high expression of CDCA3 in four subtypes of breast cancer, which were invasive ductal breast carcinoma, intra-ductal cribriform breast adenocarcinoma, invasive breast carcinoma, and invasive lobular breast carcinoma, with the highest expression-level fold-change of 4.05-times higher expression in invasive ductal breast carcinoma tissue (Figure 2B). The CDCA4 mRNA expression level in breast cancer subtypes was not as high as that of the CDCA3 level. The highest CDCA4 fold-change was 2.25-times higher relative to the control type-matched tissue (Figure 2C). The CDCA5 mRNA expression level in breast cancer tissue was the highest in the invasive ductal breast carcinoma subtype, with a 5.5-fold change relative to the control tissue (Figure 2D). The CDCA7 mRNA was overexpressed in the TNBC subtype of breast cancer by more than 1.5 - fold relative to normal control sample (Figure 2E). The CDCA8 mRNA expression level was relatively high in four subtypes of breast cancer, which were male breast carcinoma, invasive ductal breast carcinoma, invasive lobular breast carcinoma, and invasive breast carcinoma, with all of these subtypes displaying more than a 3.5-fold change over the normal tissue (Figure 2F).

### Expression of the CDCA gene family in the breast cancer cell line

**Figure 3:**
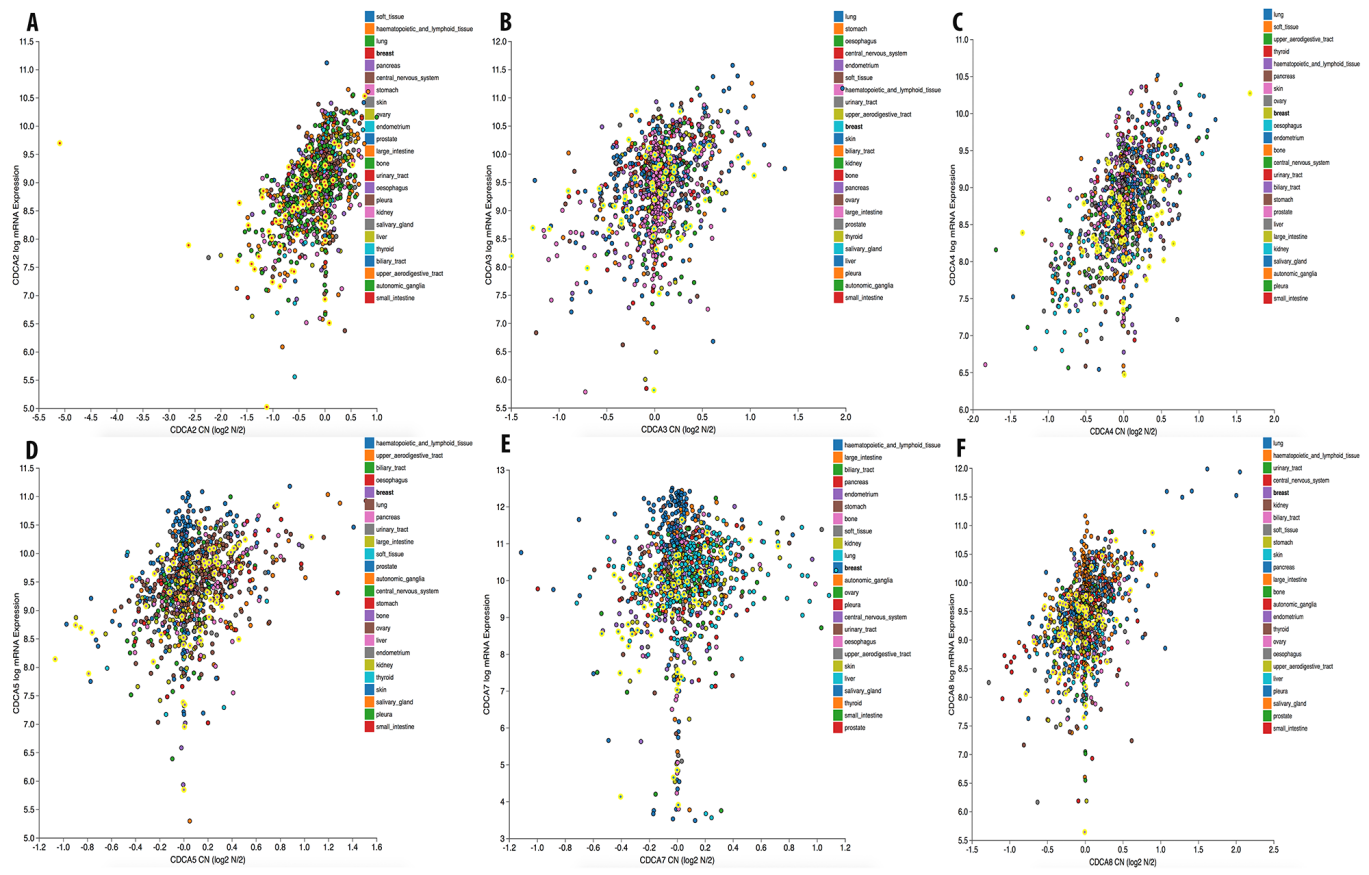
Gene expression and copy number of CDCA genes in various cancer cell lines mRNA expression and copy number of CDCAs **(A)**, CDCA3 **(B)**, CDCA4 **(C)**, CDCA5 **(D)**, CDCA7 **(E)**, and CDCA8 **(F)** in breast cancer cell line were highlighted in bright yellow color circles.

**Figure 4:**
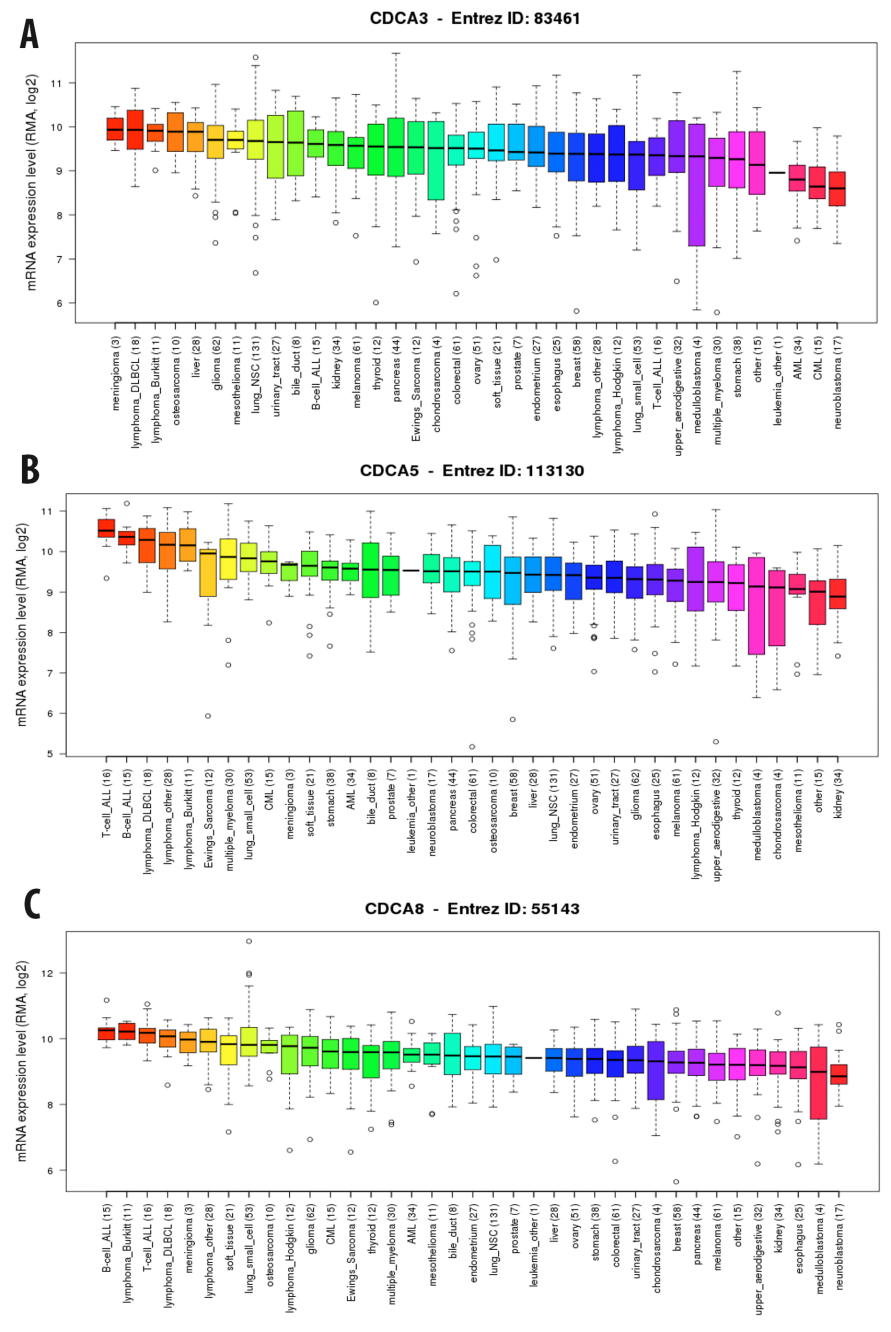
Expression of CDCA3 **(A)**, CDCA5 **(B)**, and CDCA8 **(C)** genes in breast cancer cell line from CCLE database. RMA, log2 was used to measure the expression of these three gene in breast cancer cell line.

**Figure 5:**
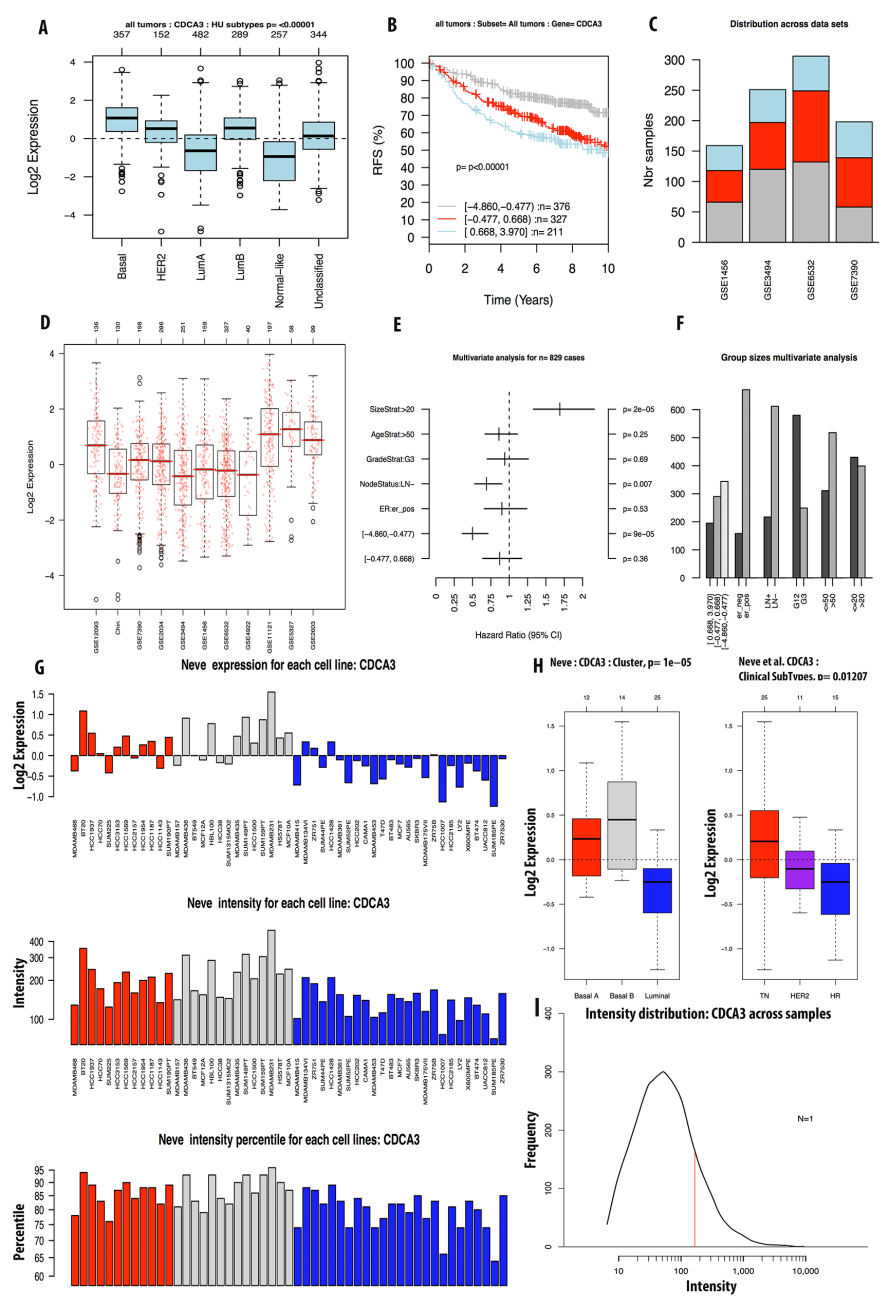
Analysis of CDCA3 expression in breast cancer tumor and cell line by GOBO database Expression of CDCA3 in six subtypes of breast cancer tumors **(A)**. RFS analysis of CDCA3 expression in tumor of patient survival with high expression in light blue, intermediate expression in red, and low expression in grey color **(B)**. CDCA3 expression across datasets **(C, D)**. Forest plot and bar chart of multivariate analysis on the expression of CDCA3 was displayed in **(E, F)**. Expression of CDCA3 in breast cancer cell line using Neve et.al dataset was display in **(G-I)**.

We continued investigating the expression of CDCA2 in cancer cell data using the CCLE database. On a log2 scale, the breast cancer cell line data from 58 datasets showed that CDCA2 expression was significantly up-regulated by an estimated eight-fold. The copy number of the CDCA2 gene in the different carcinoma cell lines is displayed in Figure 3A. The mRNA expression and copy number of CDCA3 in various cancer cell lines is displayed in Figures 3B and 4A. Neve dataset analysis of breast cancer cell lines showed the expression of CDCA3, and intensity percentile for each cell line [14]. The log2 expression levels of the six subtypes of breast cancers with up-regulated CDCA3 in basal, luminal, TN, HER2 subtypes as illustrated in Figure 5. The basal subtype had the highest expression level compared to the basal and luminal-like subtypes, whereas TN had highest expression level relative to the HER2 and hormone receptor subtypes. The highest mRNA expression of CDCA3 in breast cancer tumors was in the basal breast cancer patients. The RFS of patients with a medium expression level of CDCA3 showed an association to all tumor subtypes. The number of breast cancer samples with a high, medium, and low expression level of CDCA3 was presented in the Figure 5. The expression of all samples in various breast cancer datasets was displayed. Forest plots of 751 cases of breast cancer showed a hazard ratio (HR) for different parameters, such as tumor size, age, tumor grade, node status, and ER-positive status (Figure 5).

From the CCLE analysis, CDCA4 expression levels in breast cancer cell lines were high. The copy number of this gene was also lower than that of other cancer types (Figure 3C). The analysis of the GOBO database in tumor mode showed that log2 expression levels of CDCA4 were altered between cell lines, but not in a specific manner. In clinical subtypes, CDCA4 expression was the highest in TNBC and the lowest in HER2. Basal subtype had the highest expression of CDCA4, while the lowest was in the luminal-like subtype of breast cancer (Supplementary Figure 1).

The copy number of CDCA5 was also high in breast and other cancers (Figure 3D). The CCLE analysis of CDCA5 in cancer cell lines revealed a high expression of this gene, at greater than nine-fold compared to normal control sample (Figure 4B).

**Figure 6:**
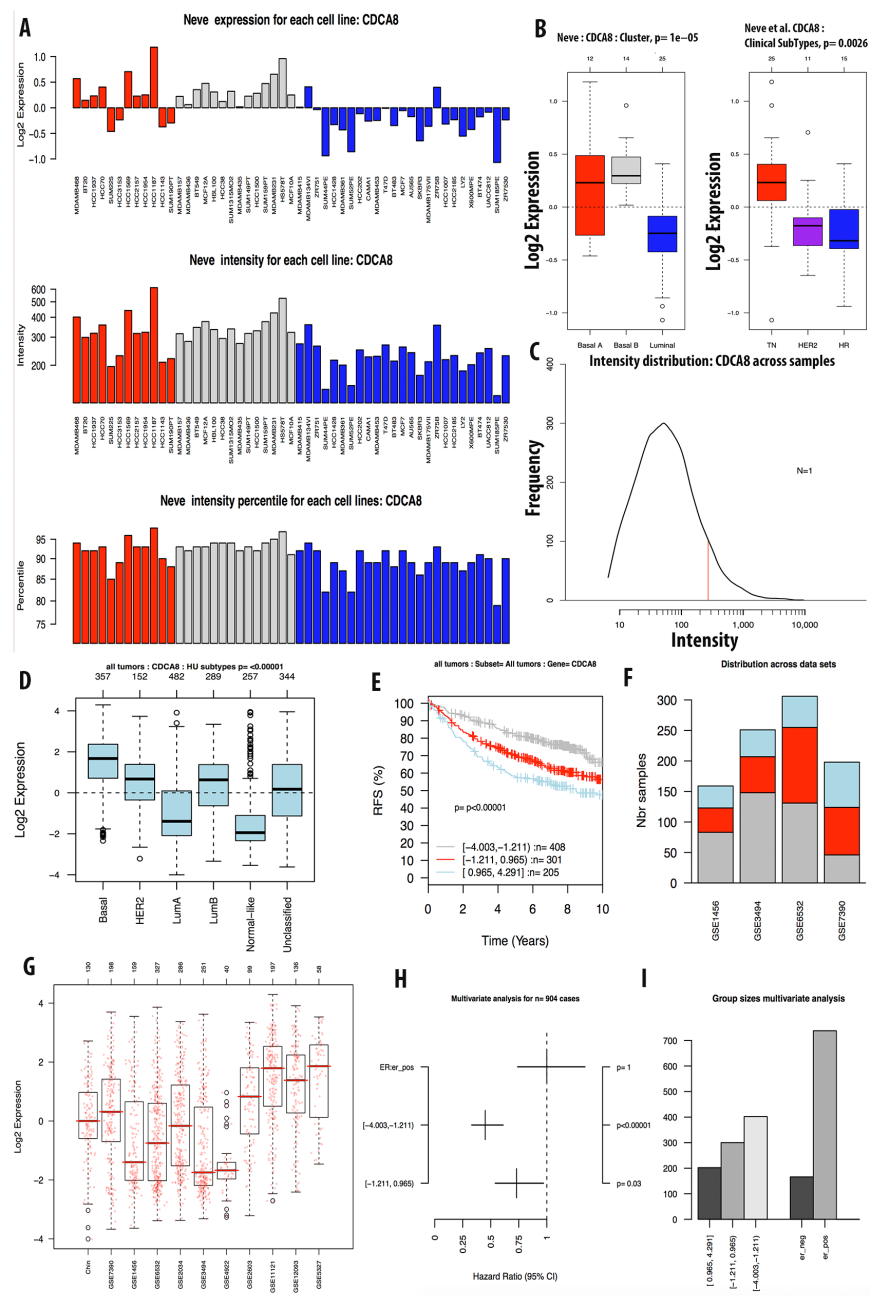
Analysis of CDCA8 expression in breast cancer tumor and cell line by GOBO database Expression of CDCA8 in six subtypes of breast cancer tumors **(A)**. RFS analysis of CDCA8 expression in tumor of patient survival with high expression in light blue, intermediate expression in red, and low expression in grey color **(B)**. CDCA8 expression across datasets **(C, D)**. Forest plot and bar chart of multivariate analysis on the expression of CDCA3 was displayed in **(E, F)**. Expression of CDCA8 in breast cancer cell line using Neve et.al dataset was display in **(G-I)**.

The expression level of CDCA7 in various cancer cell lines was ten-fold greater to control sample with a high copy number (Figure 3E). The CCLE analysis for the CDCA8 expression in different cancer cell lines showed a high expression in breast and other types of cancers (Figure 4C). The copy number of CDCA8 was also high in breast and other cancers (Figure 3F). Further analysis of the expression of CDCA8 in tumors revealed a high expression level of this gene in the basal and TNBC subtypes. The expression, intensity, and intensity percentile of CDCA8 for each cell line was analyzed using the NEVE dataset, which is also displayed in Figure 6. The RFS of patients positively correlated with CDCA8 expression. The log2 expression levels of CDCA8 in different datasets were displayed in Figure 6. The HR of the multivariate analysis for the 853 cases included tumor size, age, tumor grade, and node status of cancer patient, which are also illustrated (Figure 6).

### Expression of CDCA genes and breast cancer patient overall survival

**Figure 7:**
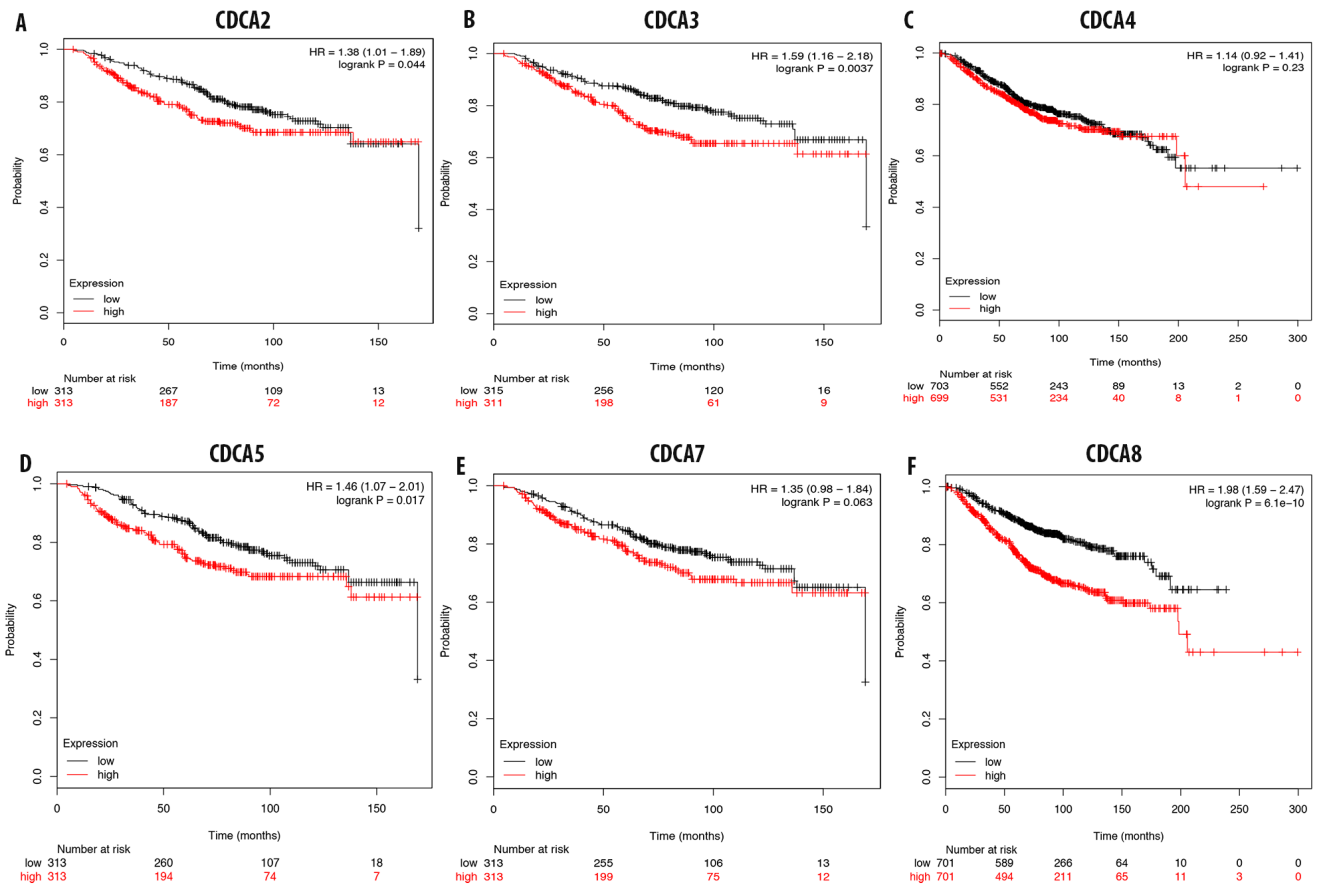
Correlation between CDCA genes **(A-F)** and overall survival of breast cancer patient. The over expression of CDCA2, CDCA3, CDCA5, and CDCA8 were highly correlated with patient survival with high HR which results in poor prognosis value. P-value<0.05 means significant different.

The overexpression of CDCA2 in breast cancer tissue was highly correlated with a poor prognosis for breast cancer patients, with an HR of 1.36 (Figure 7A). A survival analysis of breast cancer patients with an overexpression of CDCA3 showed poor prognosis (HR = 1.59) (Figure 7B). CDCD4 expression was not significantly correlated with patient survival, with an HR of 1.14 (Figure 7C). This highly expressed of CDCA5 gene results in a poor prognosis value for breast cancer patients, with an HR of 1.46, and it significantly reduced patient survival over the 3-year and 5-years interval (Figure 7D). Analysis of CDCA7 expression and breast cancer patient survival revealed no significant correlation, with an HR of 1.35 (Figure 7E). The survival analysis of breast cancer patients with high CDCA8 expression levels showed a poor prognosis value, with an HR of 1.98 (Figure 7F).

### Relapse-free survival analysis of breast cancer patients reveals a high correlation with CDCA3, CDCA5, and CDCA8

**Figure 8:**
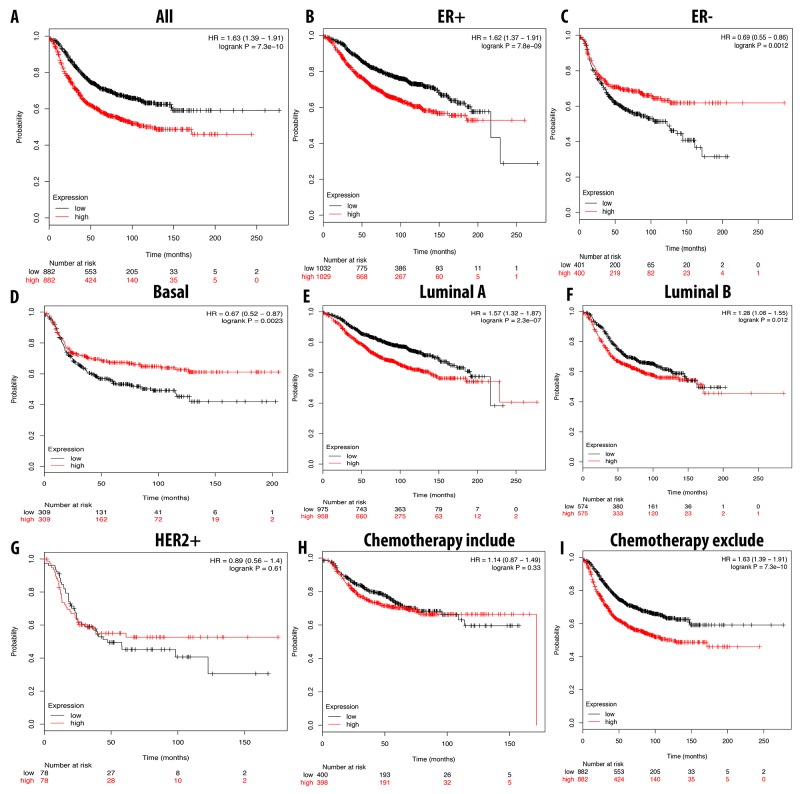
Correlation between expression of CDCA3 in RFS breast cancer patient **(A-I)**. Breast cancer patient had significant shorter RFS with high expression of CDCA3 (A). High expression of CDCA3 was significantly associated with shorter RFS in ER positive but longer RFS in ER negative (B, C). In both luminal A and luminal B, high expression of CDCA3 was significantly indicated shorter value (E, F). High expression of CDCA3 was significantly linked to longer RFS in basal subtype but not in HER2 positive (D, G). Patient underwent chemotherapy had longer RFS relative to those who did not received chemotherapy treatment (H, I). P-value<0.05 means significant different.

We further analyzed the overexpression of CDCA3, CDCA5, and CDCA8 with respect to patient RFS. In general, the high expression of these three genes resulted in a poor prognosis for the patient. A high expression of CDCA3 was highly correlated with patient survival under all breast cancer subtypes, with an HR of 1.59. In ER-positive, luminal A, and luminal B subtypes, the high expression of CDCA3 dramatically reduced the survival period for breast cancer patients. Interestingly, we found that patients undergoing chemotherapy treatment had a better prognosis relative to those who did not received this treatment, with HR values of 1.14 and 1.63, respectively. In the basal subtype, the high expression of CDCA3 was significantly correlated with a longer RFS, however in the HER2-positive subtype was not (Figure 8).

**Figure 9:**
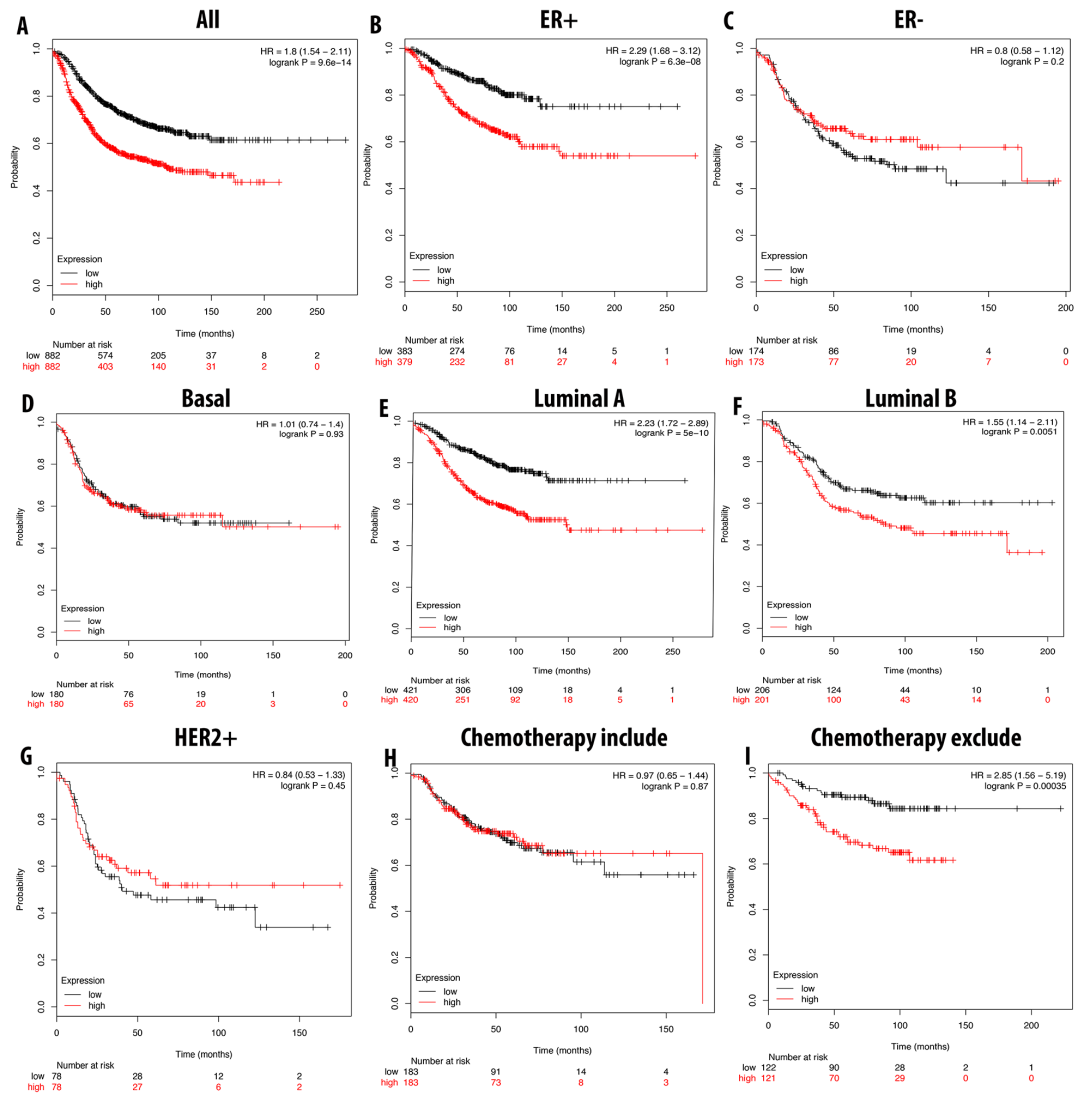
Correlation between expression of CDCA5 in RFS breast cancer patient **(A-I)**. Breast cancer patient had significant shorter RFS with high expression of CDCA5 (A). High expression of CDCA5 was significantly associated with shorter RFS in ER positive but good prognosis in ER negative (B, C). In both luminal A and luminal B, high expression of CDCA5 was significantly indicated shorter value (E, F). High expression of CDCA3 was not significantly linked to longer RFS in HER2 positive but not in basal subtype (D, G). Patient underwent chemotherapy had longer RFS relative to those who did not received chemotherapy treatment (H, I). P-value<0.05 means significant different.

A high expression level of CDCA5 was correlated with a shorter RFS in breast cancer patients overall. With ER-positive, luminal A, and luminal B subtypes, a high expression of CDCA5 significantly shortened the RFS. Furthermore, patients without chemotherapy treatments could have a 2.85 times shorter RFS compared to the ones with chemotherapy (Figure 9).

**Figure 10:**
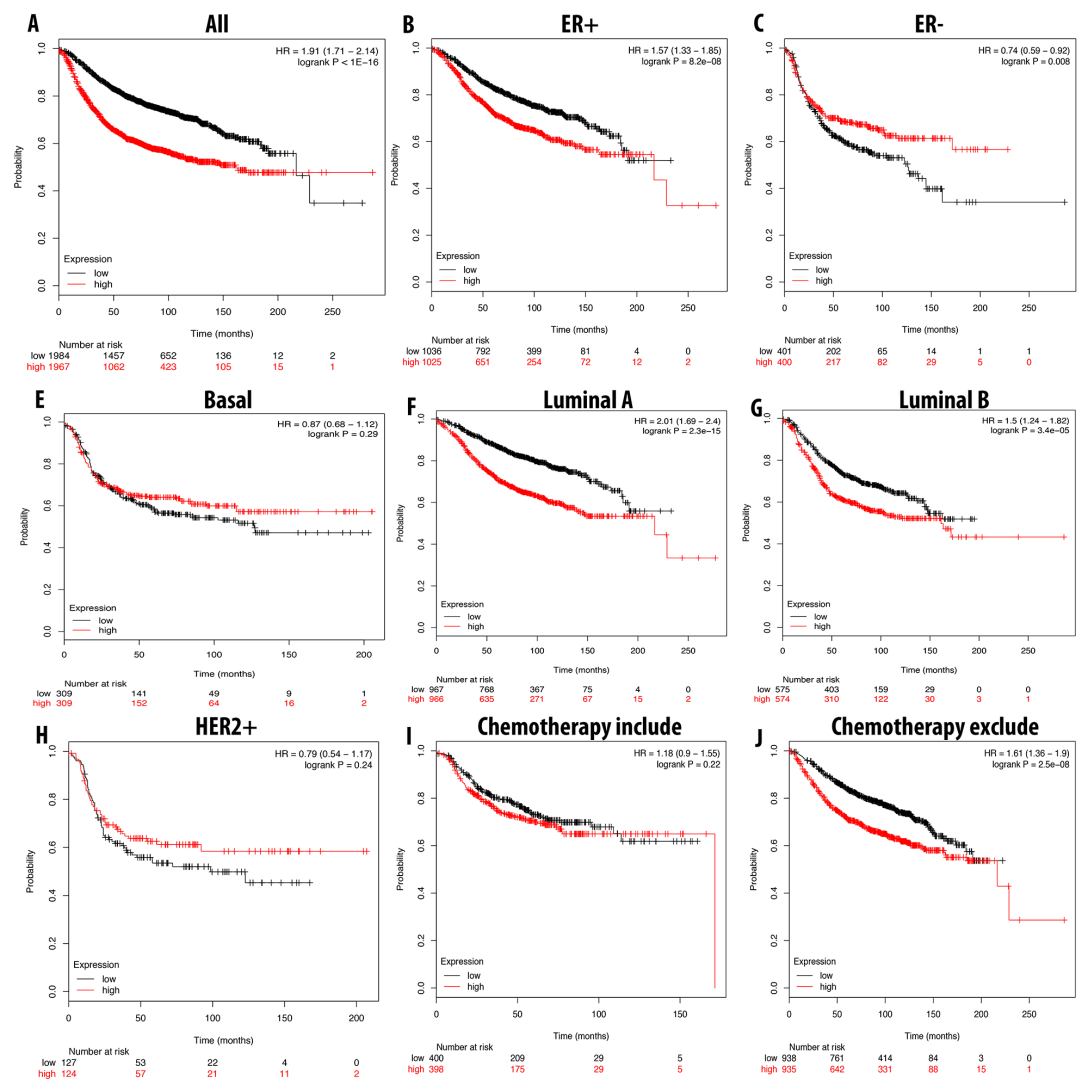
Correlation between expression of CDCA8 in RFS breast cancer patient **(A-I)**. Breast cancer patient had significant shorter RFS with high expression of CDCA8 (A). High expression of CDCA8 was significantly associated with shorter RFS in ER positive but longer in ER negative (B, C). In both luminal A and luminal B, high expression of CDCA8 was significantly indicated shorter value (E, F). High expression of CDCA8 was significantly linked to longer RFS in basal subtype and HER2 positive (D, G). Patient underwent chemotherapy had longer RFS relative to those who did not received chemotherapy treatment (H, I). P-value<0.05 means significant different.

In a similar expression pattern to CDCA3 and CDCA5, a high expression level of CDCA8 also correlated with a bad prognosis for breast cancer patients with a shorter RFS. In patients with luminal A, luminal B and ER-positive subtypes, a shorter RFS was highly correlated with CDCA8 overexpression. Patients undergoing chemotherapy treatment could prolong RFS, but not significantly (Figure 10).

### Co-expression analysis of CDCA3, CDCA5, and CDCA8 revealed their expression networks in breast cancer

**Figure 11:**
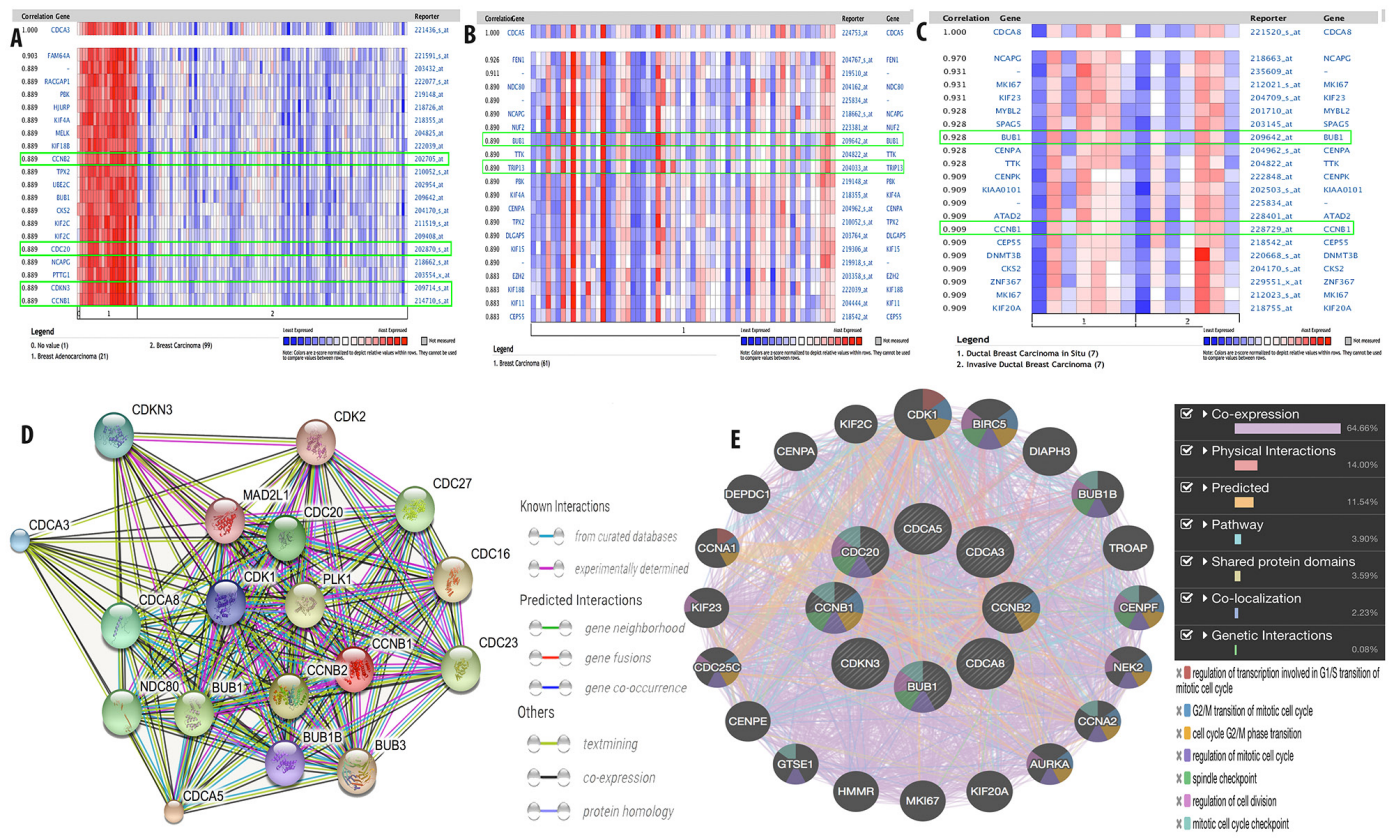
Co-expression analysis of CDCA3, CDCA5, and CDCA8 with other gene such as CCNB2, CDC20, CDKN3, CCNB1, BUB1, and TRIP13 in breast cancer **(A-C)**. Functional proteins association network of CDCA3, CDCA5, CDCA8 with their co-expressed genes **(D)** and function **(E)** was done by STRING database and GENEMANIA.

To further investigate the expression network for CDCA3, CDCA5, and CDCA8, co-expression analysis using clinical specimens was done with the Oncomine database. We found that CDCA3 expression was highly correlated with four genes, namely Cyclin B2 (CCNB2) (R=0.89), Cell Division Cycle 20 (CDC20) (R=0.89), Cyclin Dependent Kinase Inhibitor 3 (CDKN3) (R=0.89), and Cyclin B1 (CCNB1) (R = 0.89; Figure 11A). CDCA5 expression was correlated with two genes, namely budding uninhibited by benzimidazoles 1 (BUB1) (R=0.89) and Thyroid Hormone Receptor Interactor 13 (TRIP13) (R = 0.89; Figure 11B), while CDCA8 expression was correlated with BUB1 (R = 0.928) and CCNB1 (R = 0.909; Figure 11C).

The interaction network for CDCA3, CDCA5, and CDCA8 with their co-expressed genes was plotted using the STRING database for gene interaction (https://string-db.org) and GeneMANIA for gene networking and the prediction of gene function. We established the functional protein interaction network for CDCA3, CDCA5, CDCA8, CCNB2, CDC20, CDKN3, BUB1, TRIP13, and CCNB1 by using known and predicted interactions (Figure 11D). The interaction of these genes was expanded to other related genes in their network through the use of the following additional traits: co-expression, physical interactions, pathways, shared protein domains, co-localization, and genetic interactions (Figure 11E).

## DISCUSSION

Breast cancer has been found to be correlated with mutations and/or the overexpression of oncogenic genes. Finding new targets for breast cancer, particularly in specific subtypes, is extremely important for the prognosis and potential cure of this disease. In the present study, we found three members of the cell division cycle-associated gene family that had distinct mRNA expression in breast cancer tumors and cell lines, which are CDCA3, CDCA5, and CDCA8. The overexpression of these three genes correlated to the survival probability for a breast cancer patient in terms of the three-year and five-year survival periods. By analyzing the various tumor subtypes and cell lines of breast cancer, we found evidence for CDCA3, CDCA5, and CDCA8 involvement in breast cancer, resulting in an overall poor prognosis. Further analysis of patient RFS with different subtypes of breast cancer revealed that patients with different intrinsic subtypes had poorer prognoses with a high HR.

CDCA3plays an important role as a mitosis entry 1 trigger and controls cell cycle progression. Previously, CDCA3 was known to be involved in several types of cancer, such as prostate cancer, liver cancer, and oral squamous cell carcinoma [15–18]. In another bioinformatics study of 2158 full cancer transcriptomes from 163 diverse entities, CDCA3 was proven to be a novel gene involved in liver carcinogenesis [19]. In the current analysis, CDCA3 expression levels were high in invasive ductal breast carcinoma and were highly correlated with a low survival probability for breast cancer patients, leading to a poor prognosis. Our data revealed similar CDCA3 expression patterns to previous studies using whole transcription profiles of invasive ductal breast carcinoma obtained by either microarrays or RNA-sequence data. According to the previous studies, the overexpression of CDCA3 in invasive ductal breast carcinoma was likely to associate with oral carcinogenesis by decreasing the levels of cyclin-dependent kinase inhibitors, which resulted in cell cycle arrest at G1 [20, 21]. Altogether, the overexpression of CDCA3 is likely associated with cell cycle arrest at the G1 phase, a critical checkpoint for cell division, resulting in a chain reaction of descending processes that likely leads to the development of cancers. In addition, the survival analyses revealed that the expression of CDCA3 in many breast cancer subtypes highly correlated with bad prognoses. In short, CDCA3 could be considered as a potential target for these breast cancer subtypes.

CDCA5is also considered as oncogene since its overexpression has been found in many types and subtypes of cancer [22–25]. CDCA5 plays a crucial role in DNA repair [22], and is involved in the process of sister-chromatid cohesion and separation [26]. A poor prognosis for non-small cell lung cancer was linked to CDCA5 overexpression [12]. In another study, CDCA5 overexpression was linked to G1-S transition malfunction in urinary bladder urothelial carcinoma [22]. Furthermore, a method for lung cancer and/or esophageal cancer treatment and prevention based on the overexpression of CDCA5 was developed and patented, proving the possible application of CDCA5 to cancer therapeutics [24]. In this study, we found CDCA5 overexpression dramatically decreased the survival probability of breast cancer patients to lower that 0.5 in the three-year survival rate. In addition, RFS patients with different subtypes of breast cancer, ER-positive, luminal A, and luminal B, had a poorer prognosis. On the whole, CDCA5 could be considered as a target for breast cancer, particularly invasive ductal breast carcinoma.

CDCA8, a regulator of cell mitosis, was shown to be associated with lung cancer when it was phosphorylated at four positions, Ser^154^, Ser^219^, Ser^275^, and Thr^278^, by aura kinase B [27]. One meta-analysis using public microarray data and immunohistochemistry revealed that the overexpression of CDCA8 in breast cancer, especially TNBC, reduced patient survival [28]. Our data showed that CDCA8 had a high expression level in the male breast carcinoma, invasive lobular breast carcinoma, invasive ductal breast carcinoma, and invasive breast carcinoma subtypes. Moreover, highly expressed CDCA8 was also associated with an extremely low survival probability and a poor prognosis for patients with a probability lower than 0.4 at the five-year interval. RFS patients with ER-positive, luminal A, and luminal B subtypes had a poorer prognosis than the other subtypes. Thus, these data suggested the potential role of CDCA8 as a treatment target in these subtypes. In conclusion, three members of the cell division cycle-associated gene family, CDCA3, CDCA5, and CDCA8, displayed distinct overexpression in breast cancer in both tumors and cancer cell lines, and this overexpression was associated with a poor prognosis for the breast cancer patient with a low survival probability. These three genes could be considered as potential targets for breast cancer treatment and prevention.

## MATERIALS AND METHODS

### Oncomine database analysis

The CDCA family mRNA expression level was analyzed by the Oncomine database using public microarray as well as RNA-sequence database [29, 30]. This method has been clearly described in our previous studies [31–34]. Briefly, the names of the CDCA genes (CDCA2, CDCA3, CDCA4, CDCA5, CDCA7, and CDCA8) were keyed into the search box with the threshold for the p-value set to < 0.001, the fold change > 1.5, and the gene rank percentage < 10% when comparing cancerous tissue with normal type-matched tissue. Co-expression analysis was performed with clinical breast cancer samples. All calculations were set to the default settings, including the p-value, the two-sided *t*-test to compare the mean mRNA expression level between the cancer and control groups, and the multiple testing correction for the p-value to avoid the false discovery of genes with a small p-value that were not significant.

### Gene expression-based outcome for breast cancer online (GOBO) database analysis

The mRNA expression level of the CDCA gene family, specifically in breast cancer, was analyzed using the GOBO database [35]. The GOBO database allows users to perform a gene set analysis in four modes, a tumor mode, a cell line mode, a co-expressed gene mode, and a sample prediction mode. In the present study, we applied the tumor and cell line modes for the analysis of our target genes. In the tumor mode, we used the default setting from GOBO for recurrence-free survival (RFS) as the end-point of the Kaplan-Meier survival analysis and used the estrogen receptor (ER)-status for multivariate parameters. In the cell line mode, we used the breast cancer cell line from Neve et al. database [14].

### The cancer cell line encyclopedia (CCLE) database analysis

Transcriptomic expression levels of the CDCA gene family across multiple types of cancer cell lines were analyzed using the CCLE database [36]. From the CCLE, two types of data could be acquired, namely gene expression and copy number in the datasets. Gene expression in the dataset provides information about an mRNA expression level in robust multichip average (RMA) log2 form across all of the cancers from the CCLE database. Meanwhile, the gene expression and copy number in the datasets of genes from different types of cancer such as liver and lung cancer can also be acquired from CCLE database.

### Kaplan-meier plot database analysis for survival probability

The correlation between mRNA expression levels of the CDCA gene family and the survival probability of breast cancer patients was analyzed using the Kaplan-Meier plot database as previously described [16]. In brief, we input the gene name of the CDCA family into the gene symbol search box and adjusted the survival type to RFS. We kept all of the default settings of the Kaplan-Meier plot database, such as the ER status, human epidermal growth factor receptor 2 (HER2) status, intrinsic subtype, and chemotherapy status, then plotted the Kaplan-Meier curve [37].

### STRING database for functional protein association network

The Search Tool for the Retrieval of Interacting Genes (STRING) database (https://string-db.org) was used to create a network of the protein interactions for the interested genes [38]. We used the multiple protein to input data and chose *Homo sapiens* as the data source. All default settings were kept for further analysis.

### GeneMANIA database for predicting the target gene function

Gene-association networks were made for CDCA3, CDCA5, and CDCA8 using GeneMANIA (http://genemania.org) [39]. The input genes were in stripped circles. The various proteins were colored based on their involvement in specific processes, such as “regulation of transcription involved in G1/S transition mitotic cell cycle”, “G2/M transition of mitotic cell cycle”, “regulation of mitotic cell cycle”, “spindle checkpoint”, “regulation of cell division”, and “mitotic cell cycle checkpoint”. The gene interaction network was created by co-expression, physical interaction, predicted interaction, pathway, shared protein domains, co-localization, and genetic interactions.

## SUPPLEMENTARY MATERIALS AND FIGURES



## Abbreviations

CDCA: Cell division cycle-associated protein
CCLE: Cancer Cell Line Encyclopedia Database
GOBO: Gene expression-based Outcome for Breast Cancer Online database
TCGA: The Cancer Genome Atlas
DMFS: Distant metastasis free survival
RFS: Relapse free survival
(sometimes abbreviated; TNBC): Triple-negative breast cancer
ER: Estrogen receptor
HR: Hormone receptor
HER2: Human epidermal growth factor receptor 2
