# Supplementary material for: Distinct expression of CDCA3, CDCA5, and CDCA8 leads to shorter relapse free survival in breast cancer patient
Source: Oncotarget. 2018 Jan 9;9(6):6977–92. doi: 10.18632/oncotarget.24059 (PMC5805530; doi:10.18632/oncotarget.24059)
Supplement: Supplementary file 1 [file oncotarget-09-6977-s001.pdf]

Distinct expression of CDCA3, CDCA5, and CDCA8 leads to shorter relapse free survival in breast cancer patient

SUPPLEMENTARY MATERIALS

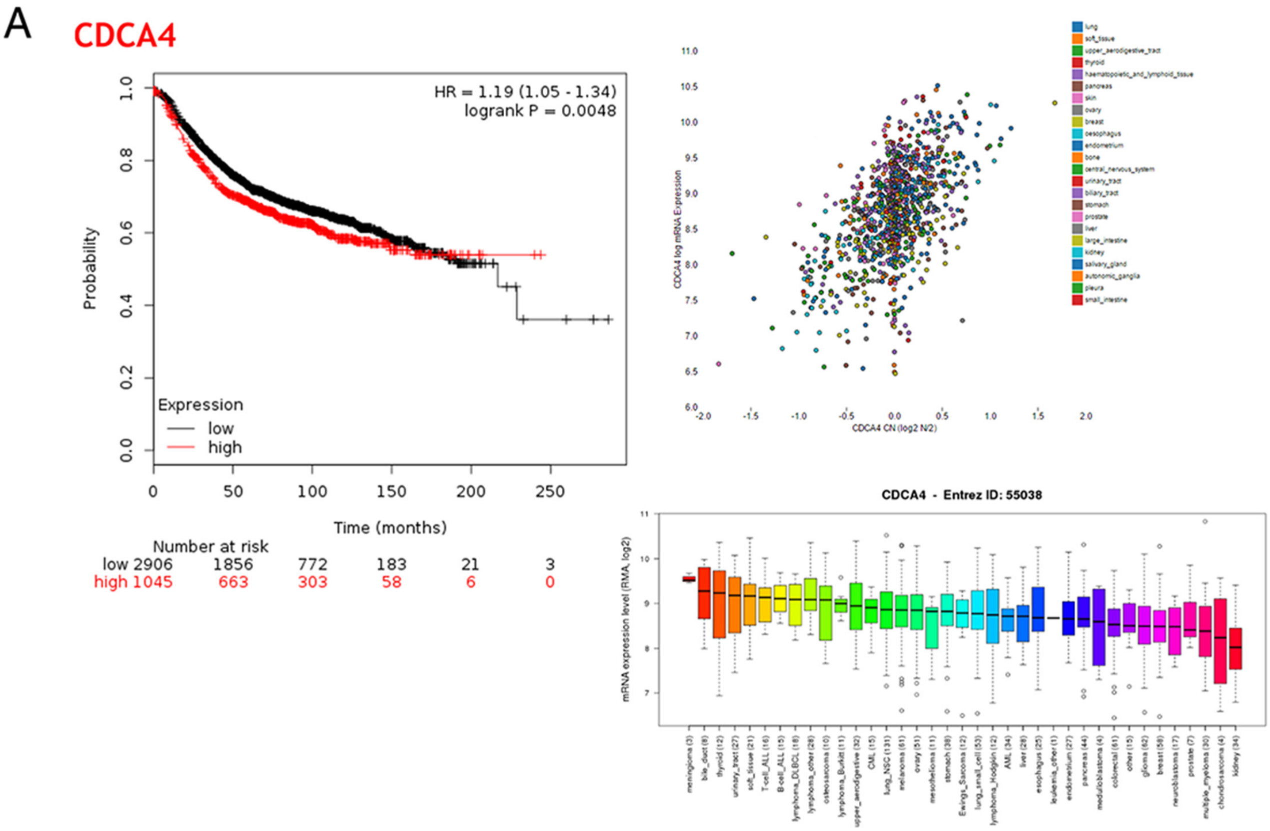

Supplementary Figure 1: CDCA4 mRNA expression level and breast cancer patient survival, copy variation number of CDCA in multiple types of cancers. mRNA expression level of CDCA4 in cancer cell lines **(A)**.

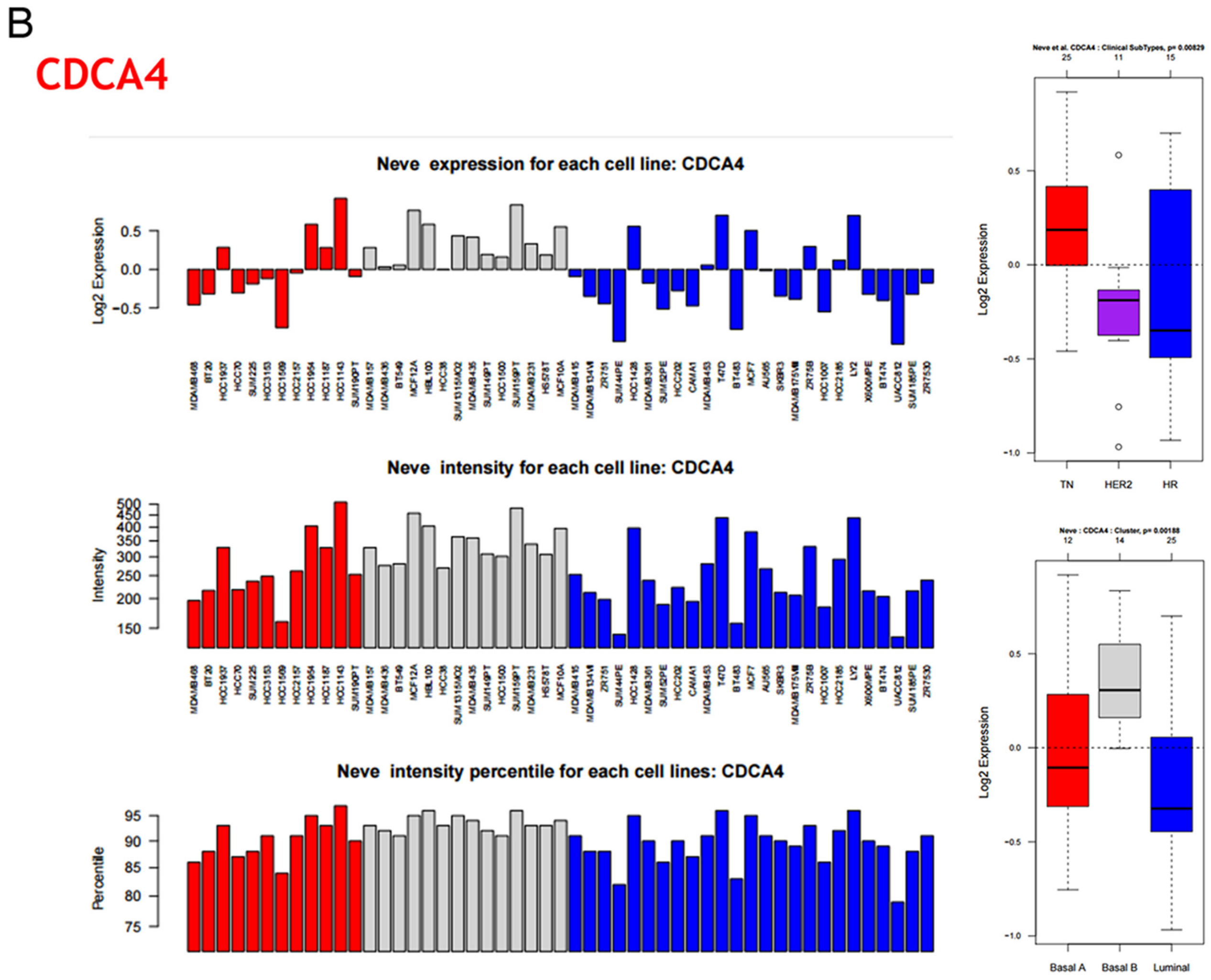

Supplementary Figure 1: (*Continued*) CDCA4 mRNA expression level and breast cancer patient survival, copy variation number of CDCA in multiple types of cancers. mRNA expression of CDCA4 in Neve breast cancer dataset **(B)**.

C

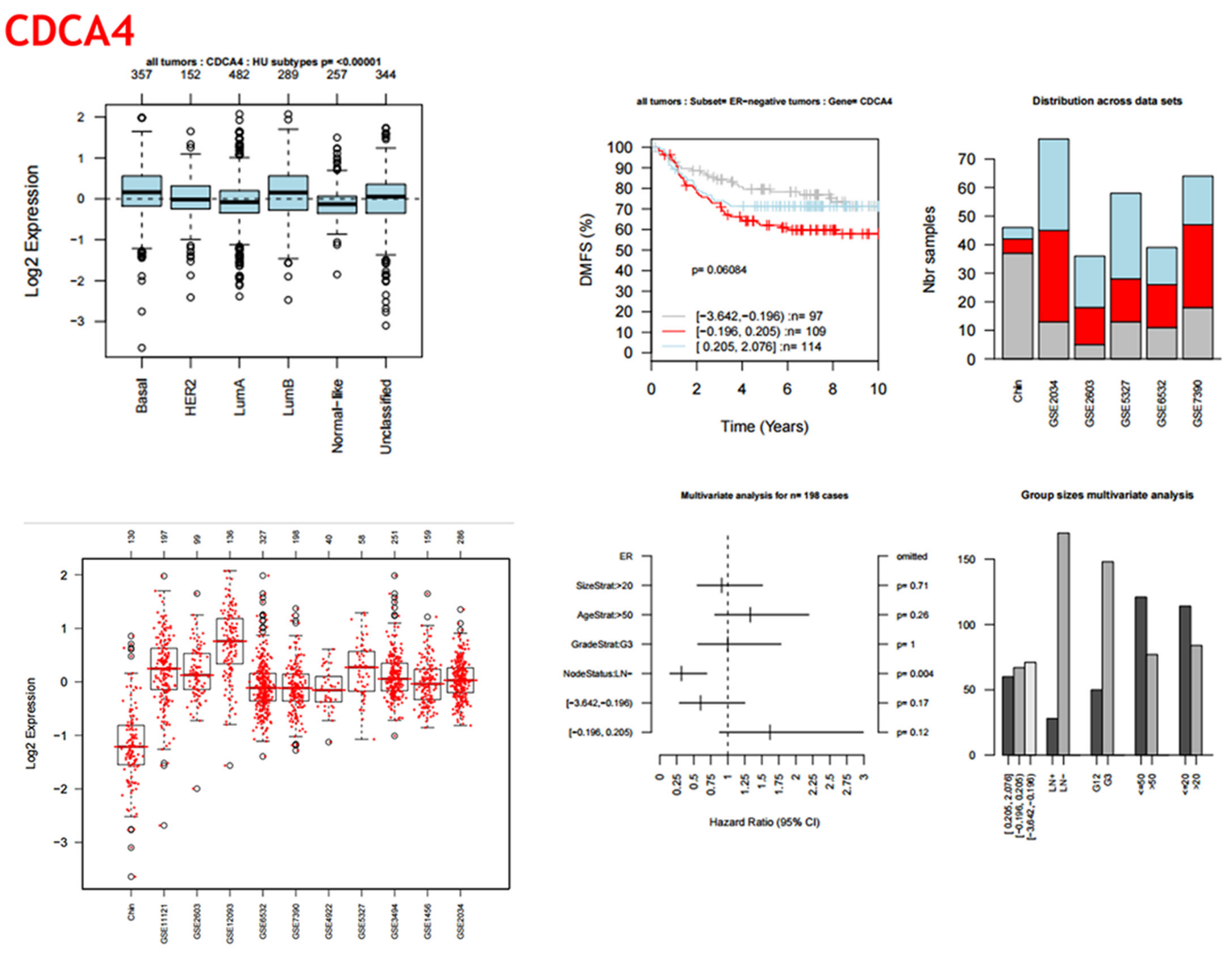

Supplementary Figure 1: (*Continued*) CDCA4 mRNA expression level and breast cancer patient survival, copy variation number of CDCA in multiple types of cancers. Expression of CDCA4 by GOBO database analysis **(C)**.
